# Sleep medication use and risk of fractures in breast cancer survivors

**DOI:** 10.1007/s10549-021-06392-4

**Published:** 2021-09-29

**Authors:** Reina Haque, Rowan T. Chlebowski, LieHong Chen

**Affiliations:** 1 Department of Research and Evaluation, Kaiser Permanente Southern California, 100 South Los Robles, 2nd Floor, Pasadena, CA 91101 USA; 2 Department of Health Systems Science, Kaiser Permanente Bernard J. Tyson School of Medicine, Pasadena, CA 91101 USA; 3 Lundquist Research Institute, Harbor-UCLA Medical Center, Torrance, CA USA

**Keywords:** Breast cancer, Sleep medication, Fractures, Comorbidity

## Abstract

**Purpose:**

Sleep problems are more common in breast cancer survivors than those without a cancer history. Our goal was to examine the risk of fractures among breast cancers survivors who used prescription sleep aids.

**Methods:**

We conducted a retrospective cohort study of 21,346 adult women diagnosed with stage 0–III breast cancer between 2009 and 2016 and followed them through 2017. We examined person-year rates of fractures by sleep medication use and calculated adjusted hazard ratios (HR) and 95% confidence intervals (CI) with Cox proportional hazards models using time-dependent variables for sleep medications and covariate medications (antidepressants, anti-anxiety medications, and bisphosphonates) adjusted for demographics, comorbidities, and tumor characteristics and cancer treatments.

**Results:**

The sleep medication use was common (40%) in breast cancer survivors and was associated with a 33% increased risk of fractures (adjusted HR = 1.33, 95% CI: 1.20–1.49). Further, in a sensitivity analysis based on new use of sleep medication, the fracture risk was even stronger (adjusted HR = 1.44, 95% CI: 1.26–1.64).

**Conclusion:**

Given the high use of sleep medications and the high risk of fractures in breast cancer survivors, this study suggests that non-pharmacologic management of sleep problems might be considered as alternative therapy.

**Supplementary Information:**

The online version contains supplementary material available at 10.1007/s10549-021-06392-4.

## Introduction

For many of the 3.8 million female breast cancer survivors living in the US in 2019, improved survival is complicated by long-term psychosocial effects including sleep problems [1]. The prevalence of insomnia symptoms is nearly 40% in cancer survivors versus 10%–15% in the general U.S. population [2]. Studies suggest that sleep problems arise due to the side-effects of cancer treatments or due to the disease itself [3] and can persist years after primary cancer therapy completion [4]. Breast cancer survivors are more likely to have physical symptoms and psychological distress than patients with other types of cancers possibly due the treatment side-effects [5, 6]. For example, hot flashes caused by tamoxifen can lead to or exacerbate sleep problems in breast cancer survivors [7]. However, limited information exists about use of sleep aids in breast cancer survivors and consequent health outcomes.

Prescription sleep aids include benzodiazepines, non-benzodiazepines acting on benzodiazepine receptors, and antidepressants. Furthermore, new research suggests the risk of hip fractures rises soon after an older person is prescribed a sleeping pill [8]. Given this background, breast cancer survivors may have an even greater overall risk of fractures due to the bone loss induced by adjuvant hormonal therapy (aromatase inhibitors), toxicity of the chemotherapies themselves, and/or greater use of sleep aids to cope with sleep problems.

Previous studies that examined the adverse health effects of sleep medications in cancer survivors were limited because they included data from a single academic hospital, were based on patient with various types of cancer, or did not address confounding factors such as comorbidities or use of antidepressants [9–13]. In sum, sparse population-based data exist about these serious sequelae of prescription sleep medication use in breast cancer survivors. Thus, our objective was to examine fracture risk in breast cancer survivors who used sleep medications, while accounting for antidepressant use, psychosocial status, osteoporosis, other comorbidities, tumor characteristics, adjuvant cancer, and bisphosphonates (bone enhancing drugs). This knowledge can inform interdisciplinary clinical management of tens of thousands breast cancer survivors experiencing sleep problems as result of their disease, side-effects of cancer treatments, or distress.

## Methods

### Data sources and setting

This study was conducted at Kaiser Permanente Southern California (KPSC), a large managed care system that comprises nearly 15 hospitals and over 200 medical clinics that serve with 4.7 million members. Patients receive virtually all their medical care and prescription drugs within this integrated healthcare delivery system, and information on any outside procedures and diagnoses are available through claims databases. The health plan’s National Cancer Institute-Surveillance, Epidemiology, and End Results (SEER)-affiliated tumor registry was used to identify subjects with breast cancer. The KPSC Institutional Review Board reviewed and approved this study.

### Subjects and design

We assembled a cohort of women diagnosed with first primary breast cancer in 2009–2016 followed through December 2017. Eligible women included adults (> 18 years at diagnosis), with American Joint Commission on Cancer TNM Stage 0-III breast cancer, and with at least one year of continuous membership prior to their cancer diagnosis (*n* = 21,513). We excluded 167 women with a history of fractures in the prior 3 months before breast cancer diagnosis to reduce confounding, leaving 21,346 for analysis.

### Incident fractures outcomes

We identified first incident fractures that occurred after breast cancer diagnosis, from the health plan’s electronic health records (EHR). Fractures of the forearm, femur, lower leg, wrist and hand, vertebrae, and pelvis were identified using ICD9 (International Classification of Diseases, Ninth Revision) codes and ICD10 codes (see Supplement Table S1). The event that occurred first served as the outcome.

### Sleep medications

The pharmacy dispensing database was used to capture use of all sleep aids (name, date of initiation, days supplied) in the health plan’s formulary: lorazepam, trazodone, doxepin, flurazepam, temazepam, triazolam, eszopiclone, zaleplon, zolpidem, and suvorexant. We also considered the effect of “new use” of the sleep medication on risk of fracture. “New use” was defined as the drug dispensing that occurred without drug possession in the preceding 3 months before breast cancer diagnosis.

### Covariates

A comprehensive set of covariates was captured from the EMR. These included race/ethnicity and tumor factors (age and stage at breast cancer diagnosis, adjuvant cancer therapy, diagnosis year, and tumor characteristics). We also captured prior comorbidities from one year before breast cancer diagnosis, and current comorbidities post breast cancer diagnosis through the end of each woman’s follow-up. Current comorbidities ascertained included osteoporosis, bone mineral density, hypertension, depression, dementia, anxiety, and sleep problems. We extracted data on covariate medications such as antidepressant, anti-anxiety, and bisphosphonate use during the follow-up period. Bisphosphonates (alendronate, which was the most commonly used drug in 93% of patients) was prescribed to combat the bone loss typically associated with adjuvant aromatase inhibitor therapy.

### Statistical analyses

Follow-up commenced on the breast cancer diagnosis date and censored on the date of the earliest study endpoint: first fracture diagnosis date, death, termination of health plan membership, or study’s end (December 31, 2017). The definition of continuous health plan enrollment allowed for gaps of up to 3 months in enrollment during study period, as these were likely administrative gaps. In descriptive analyses, we compared the distribution of all variables (demographics, tumor characteristics, comorbidities, and covariate drugs) by fracture status and sleep medication use. The Chi-square or Fisher exact tests were used to compare categorical variables, and the Kruskal–Wallis tests was used for continuous variables. Because women were followed different lengths of time, we computed the person-year rates of fractures. Odds ratios and 95% confidence intervals (CI) were used to compute the association of mental health conditions with use of sleep medications and correlations between sleep medications and psychiatric medications.

Crude and adjusted hazard ratios (HR) and 95% CIs were estimated for fracture risk by Cox proportional hazards models using time-dependent variables for sleep aids and the other covariate medications (antidepressants, anti-anxiety medications, bisphosphonates) used during the follow-up period. We also adjusted for adjuvant endocrine therapy (tamoxifen and aromatase inhibitors treated as time-dependent) in the models. All covariates selected for adjustment in the model were based on clinical importance and descriptive statistics. The proportional hazards assumption was tested via graphic plots and Schoenfeld residuals; no violations were found.

We also conducted additional analyses to assess the robustness of the multivariable results of the association between sleep medication use and fracture risk. First, we evaluated two models stratified by bisphosphonate use status using the full cohort (*n* = 21,346). Second, we repeated analyses on the subset of women who were “new users” of sleep medications (*n* = 16,486). Third, we conducted a sensitivity analysis based on bone mass density (BMD) data during study follow-up; this was available on *n* = 8,498 survivors with spine BMD; if multiple BMD data were available per patient, we use the lowest to be conservative. Except for body mass index (BMI, < 5% was missing), missing data were rare for the study variables. Unknown BMI was entered as a category in the multivariable models (as done for race/ethnicity which had 1% missing in the other/mixed/unknown category). All analyses were performed using SAS Version 9.4 (SAS Institute, Cary NC).

## Results

In the cohort 21,346 breast cancer survivors, we observed 2,038 incident fractures during the 85,425 person-years of follow-up (median of 3.6 years [interquartile range, IQR: 2.0–5.9]). The median age at breast cancer diagnosis was 62.0 years (IQR: 53.0–70.0). The cohort was diverse with 51.9% non-Hispanic White; 20.4% Hispanic; 13.2% African American/Black; 13.5% Asian/Pacific Islanders; and 1.1% of mixed/unknown race/ethnicity (Table 1). Those who suffered fractures were more likely to be aged > 65 years, non-Hispanic White, and have comorbid depression, anxiety, dementia or sleep problems compared to those who did not have fractures (P < 0.001 for all variables). Those who experienced fractures were also more likely to have used antidepressants (32.4%) than those who did not have fractures (23.2%).

**Table 1:** Characteristics of breast cancer survivors by incident fracture status

	Fractures			
	No	Yes	Total	
	(*n*=19,308)	(*n*=2038)	(*n*=21,346)	
	*n* (%)	*n* (%)	*n* (%)	*P* value^a^
Baseline Demographics (time of BC diagnosis)				
Age at breast cancer diagnose, year				<0.001
18-40	947 (4.9)	33 (1.6)	980 (4.6)	
41-64	10778 (55.8)	778 (38.2)	11556 (54.1)	
65-80	6497 (33.7)	899 (44.1)	7396 (34.7)	
80+	1086 (5.6)	328 (16.1)	1414 (6.6)	
Median (IQR)	61 (52.0, 69.0)	68 (60.0, 76.0)	62 (53.0, 70.0)	<0.001
Race/ethnicity				<0.001
Non-Hispanic White	9731 (50.4)	1340 (65.8)	11071 (51.9)	
Hispanic	4009 (20.8)	343 (16.8)	4352 (20.4)	
Black	2636 (13.7)	182 (8.9)	2818 (13.2)	
Asian/Pacific Islander	2724 (14.1)	156 (7.7)	2880 (13.5)	
Other/unknown	208 (1.1)	17 (0.8)	225 (1.1)	
BMI (kg/m^2^)				<0.001
Underweight (Less than 18.5)	227 (1.2)	29 (1.4)	256 (1.2)	
Normal (18.5 to 24.9)	5036 (26.1)	619 (30.4)	5655 (26.5)	
Overweight (25 to 29.9)	5869 (30.4)	630 (30.9)	6499 (30.5)	
Obese (30 or more)	7086 (36.7)	676 (33.2)	7762 (36.4)	
Missing	1090 (5.7)	84 (4.1)	1174 (5.5)	
Covariates up to one year before BC diagnosis				
Charlson comorbidity index, weighted, exclude breast cancer				<0.001
0	11111 (57.6)	909 (44.6)	12020 (56.3)	
1	3511 (18.2)	426 (20.9)	3937 (18.4)	
2	2170 (11.2)	261 (12.8)	2431 (11.4)	
3+	2516 (13.0)	442 (21.7)	2958 (13.9)	
Osteoporosis	1811 (9.4)	475 (23.3)	2286 (10.7)	<0.001
Hypertension	8833 (45.8)	1190 (58.4)	10023 (47.0)	<0.001
Psychologic treatments				
Sleep aid	2511 (13.0)	403 (19.8)	2914 (13.7)	<0.001
Antidepressant	3938 (20.4)	609 (29.9)	4547 (21.3)	<0.001
Anti-anxiety	333 (1.7)	73 (3.6)	406 (1.9)	<0.001
Bisphosphonates	1561 (8.1)	374 (18.4)	1935 (9.1)	<0.001
Covariates during follow-up period				
Psychologic diagnosis factors				
Depression	4763 (24.7)	819 (40.2)	5582 (26.2)	<0.001
Anxiety	6596 (34.2)	917 (45.0)	7513 (35.2)	<0.001
Dementia	526 (2.7)	227 (11.1)	753 (3.5)	<0.001
Sleep problem	3781 (19.6)	618 (30.3)	4399 (20.6)	<0.001
Psychologic treatments				
Sleep aid	7218 (37.4)	1007 (49.4)	8225 (38.5)	<0.001
Antidepressant only	4475 (23.2)	660 (32.4)	5135 (24.1)	<0.001
Anti-anxiety only	3226 (16.7)	250 (12.3)	3476 (16.3)	<0.001
Bisphosphonates	2995 (15.5%)	821 (40.3%)	3816 (17.9%)	<0.001
Follow-up, year				
Median (IQR)	3.8 (2.1, 6.0)	2.4 (1.1, 4.1)	3.6 (2.0, 5.9)	<0.001
Range	(0.0-9.0)	(0.0-8.9)	(0.0-9.0)	

In Table 2, women who had fractures were more likely to have invasive breast cancer (Stages I–III, 83.1%) than those who did not have fractures (78.8%). In terms of cancer therapy, those who underwent adjuvant chemotherapy were less likely to have fractures (*P* < 0.001), but those who used adjuvant hormonal therapy (*P* = 0.004) were more likely to experience fractures. We did not find a statistically significant difference by adjuvant radiation therapy (*P* = 0.09).

**Table 2:** Tumor characteristics & breast cancer treatments by incident fracture status

	Fractures			
	No	Yes	Total	
	(*N* = 19,308)	(*N* = 2038)	(*N* = 21,346)	
	*n* (%)	*n* (%)	*n* (%)	*P* value
Stage at diagnosis				< 0.001
Stage 0	4102 (21.3)	344 (16.9)	4446 (20.8)	
Stage I	8230 (42.6)	976 (47.9)	9206 (43.1)	
Stage II	5376 (27.8)	566 (27.8)	5942 (27.8)	
Stage III	1600 (8.3)	152 (7.5)	1752 (8.2)	
Surgery	18,674 (96.7)	1981 (97.2)	20,655 (96.8)	0.238
Adjuvant chemotherapy	6795 (35.2)	569 (27.9)	7364 (34.5)	< 0.001
Adjuvant endocrine therapy	11,409 (59.1)	1271 (62.4)	12,680 (59.4)	0.004
Adjuvant radiation therapy	8792 (45.5)	888 (43.6)	9680 (45.4)	0.090

### Sleep aid medication use

Roughly 40% of the cohort used prescription sleep aid medications (38.5%, 8225/21, 346) for a median of 60.0 days (Table 3). The most commonly used sleep medication was lorazepam (used by *n* = 5819 women), followed by trazodone (*n* = 2296), and temazepam (*n* = 1615). As shown in Table 3, women used multiple types of sleep medications. Overall, the median cumulative duration of sleep medication use was 60 days (interquartile range, IQR: 15–268 days). Sleep aid medication use was strongly correlated with anti-anxiety medication use (OR = 3.65, 95% CI: 3.36–3.97); antidepressant use (OR = 3.97% CI: 3.69–4.28); and use of both of these medications (OR = 11.07, 95% CI: 0.08–12.17). Breast cancer survivors who used sleep aid medications were more likely to have depression (40.5% vs. 17.2%, OR = 3.27, 95% CI: 3.07–3.49) and anxiety (57.3% vs. 21.4%; OR = 4.94, 95% CI: 4.65–5.25) than non-users (Table 4). The most common antidepressants used were selective serotonin reuptake inhibitors (SSRI), followed by tricyclic antidepressants (TCA), and serotonin-norepinephrine reuptake inhibitors (SNRI); most women used multiple classes of antidepressants during follow-up (Table 4).

**Table 3:** Use of sleep aid medications among breast cancer survivors during follow-up

	Sleep aid medications
	*N* = 8225
	*n* (%)
Sleep medication use*	
Doxepin	119 (1.5)
Eszopiclone	26 (0.3)
Flurazepam	12 (0.2)
Lorazepam	5819 (70.8)
Suvorexant	4 (0.05)
Temazepam	1615 (19.6)
Trazodone	2296 (27.9)
Triazolam	72 (0.9)
Zaleplon	2 (0.02)
Zolpidem	1540 (18.7)
Cumulative duration (days)	
Median (IQR)	60.0 (15.0, 268.0)

*Exceeds 100% due to use of multiple types

**Table 4:** Psychiatric medications and mental health status in breast cancer survivors during follow-up by sleep aid medication use

	Sleep aid medication use		
	No	Yes	Total
	(*N* = 13,121)	(*N* = 8225)	(*N* = 21,346)
	*n* (%)	*n* (%)	*n* (%)
Mental health conditions after breast cancer			
Depression	2255 (17.2)	3327 (40.5)	5582 (26.2)
Anxiety	2801 (21.4)	4712 (57.3)	7513 (35.2)
Psychiatric medication use			
Antidepressants only	2625 (20.0)	2510 (30.5)	5135 (24.1)
Anti-anxiety drugs only^a^	1849 (14.1)	1627 (19.8)	3476 (16.3)
Used both drugs	827 (6.3)	2205 (26.8)	3032 (14.2)
Neither	7820 (59.6)	1883 (22.9)	9703 (45.5)
Antidepressant type^b^			
MOAI only	4 (0.03)	0 (0)	4 (0.02)
NDRI only	80 (0.6)	73 (0.9)	153 (0.7)
SARI only	1 (0.01)	1 (0.01)	2 (0.01)
SNRI only	385 (2.9)	246 (3.0)	631 (3.0)
SSRI only (Paroxetine, Fluoxetine, other SSRI)	885 (6.7)	748 (9.1)	1633 (7.7)
TCA only	581 (4.4)	314 (3.8)	895 (4.2)
TECA only	61 (0.5)	58 (0.7)	119 (0.6)
Other types	21 (0.2)	24 (0.3)	45 (0.2)
Multiple types	607 (4.6)	1046 (12.7)	1653 (7.7)
Psychiatric drug use, cumulative duration (days)			
Median (IQR)			61.0 (6.0, 569.0)

^a^Includes: aripiprazole, asenapine, chlorpromazine, clozapine, haloperidol, loxapine, lurasidone, olanzapine, perphenazine, pimozide, prochlorperazine, quetiapine, thioridazine, thiothixene, trifluoperazine, ziprasidone

^b^*MOAI* Monoamine oxidase inhibitors; *NDRI* norepinephrine–dopamine reuptake inhibitor; *SARI* Serotonin antagonist and reuptake inhibitors; *SNRI* Serotonin and norepinephrine reuptake inhibitors; *SSRI* Selective serotonin reuptake inhibitors; *TCA* Tricyclic antidepressants; *TECA* Tetracyclic antidepressants

### Fracture risk

Sleep aid medication use was associated with a greater fracture rate in breast cancer survivors (28.62/1000 person-years) than for non-use (20.52/1000 person-years) (Table 5). Correspondingly, in the multivariable Cox proportional hazards model that handled medication use as time-varying, sleep aid medication use was associated with a 33% increased fracture risk (adjusted HR = 1.33 [95% CI: 1.19–1.48], *P* < 0.001) after adjusting for race/ethnicity, age, body mass index, cancer stage, adjuvant cancer therapy, year of diagnosis, osteoporosis, depression, anxiety, dementia, Charlson Comorbidity Index, and use of antidepressants and anti-anxiety medications. This association was similar after we stratified by bisphosphonate use (adjusted HR for sleep medication and fractures = 1.36 [95% CI: 1.14–1.62] for bisphosphonate use, and HR = 1.32 [95% CI: 1.14–1.51] for bisphosphonate non-use). In the second sensitivity analyses based on the subset of “new use” of sleep aid medications, anti-anxiety and antidepressants (*n* = 16,486), the association was even stronger with an adjusted HR = 1.44 [95% CI: 1.26–1.64] (Table 5). The strong association between sleep aid medications and fracture risk persisted for bisphosphonate use (adjusted HR = 1.51 [95% CI: 1.22–1.87]) and bisphosphonate non-use (adjusted HR = 1.39 [95% CI: 1.17–1.64]). In the third sensitivity analysis based on the subset of women with spine bone mineral density (n = 8,498), as expected, we found that the fracture risk associated with sleep medication use decreased with better T-scores: T-score < − 2.5 indicating osteoporosis (adjusted HR = 1.94, 95% CI: 1.39–2.69; T-scores between − 2.5 and − 1.0 indicating osteopenia (adjusted HR = 1.47, 95% CI: 1.18–1.84; and T-score > − 1.0 indicating normal (adjusted HR = 1.38, 95% CI: 1.03–1.85) (Supplement Table S2).

**Table 5:** The association of sleep medications use and risk of fractures among breast cancer survivors in based on full cohort (a) and subset of new users (b) of sleep aid medications

	Total, n	With fractures, n	Person-years	Crude rates per 1,000 person-years	Crude HR (95% CI)	Adjusted HR (95% CI)^a^	Adjusted HR (95% CI)^b^
Sleep aids							
No (reference)	13,121	1031	50,243	20.52	1.00 (ref)	1.00 (ref)	1.00 (ref)
Yes	8225	1007	35,182	28.62	1.45 (1.33–1.58)	1.33 (1.19–1.48)	1.44 (1.26–1.64)
BP only (n=3,816)							
Sleep aids							
No (reference)	2271	403	9483	42.50	1.00 (ref)	1.00 (ref)	1.00 (ref)
Yes	1545	418	7022	59.53	1.43 (1.25–1.64)	1.36 (1.14–1.62)	1.51 (1.22–1.87)
No BP (n=17,530)							
Sleep aids							
No (reference)	10,850	628	40,760	15.41	1.00 (ref)	1.00 (ref)	1.00 (ref)
Yes	6680	589	28,161	20.92	1.44 (1.29–1.62)	1.32 (1.14–1.51)	1.39 (1.17–1.64)

^a^Main model adjusted for age, race/ethnicity, BMI, osteoporosis, comorbidities, sleep problems history, history of psychiatric medications, cancer stage, cancer treatments, and antidepressants, anti-anxiety drugs, and

^b^Sensitivity analyses based on new use of sleep medications (*N*=16,486)

## Discussion

Among 21,346 breast cancer patients without prior fractures, prescription sleep aid medication use was associated with a 33% increased risk of fractures during the 85,425 person-years of follow-up (median of 4.0 years [interquartile range: 2.2–6.1 years]). Additionally, the fracture risk associated with sleep aids persisted even after we accounted for bisphosphonate use. Furthermore, in a sensitivity analysis based on breast cancer survivors with new use of sleep aid medications, the fracture risk was 44% greater. To our knowledge, our study is the first to integrate comprehensive set of covariates in examining the fractures risk related to sleep aid medication use in a large community-based sample of breast cancer survivors. Our results suggest that prescription sleep aids increase the fracture risk in this vulnerable population.

Reasons for our findings relate to the hypnotic properties of the sleep medications that linger through the day exacerbating the risk of falls. In addition, breast cancer survivors taking aromatase inhibitors (AIs) might also be vulnerable to this effect given that AI’s are associated with acceleration of bone loss and an increased risk of osteoporotic fractures. Due to the adverse bone effects associated with AIs, patients are given bisphosphonates to combat this, but our study did not find a marked protection from bisphosphonates. Similarly, other epidemiologic studies have not found a protective effect of bisphosphonates in reducing fractures risk [14–16].

Our study has several strengths. The large sample size and comprehensive healthcare coverage enabled us to examine the fracture risk without concerns about variable healthcare insurance coverage that is known to affect cancer outcomes. Importantly, our analysis was based on filled prescriptions which ensured accuracy for both exposure and covariate drug ascertainment. Further, the study had sufficient long follow-up time to identify fracture risk, with a median of 4 years, ranging up to 9 years. Finally, we applied different analytic strategies to address various sources of bias in observational studies, and we conducted sensitivity analyses to assure the robustness of the conclusion. The HR estimates in our study accounted for demographics, comorbidities, tumor characteristics, cancer treatments, and concurrent medications. In the multivariable Cox proportional hazards model, medications were handled as time-dependent thus reducing drug exposure misclassification. Moreover, our sensitivity analyses restricted to women who were new users of sleep medications; this further strengthened the finding of the association between sleep medications and fracture risk. Lastly, about half of the breast cancer survivors were from minority groups, which enhances generalizability of our results to the larger California population. Thus, our carefully designed longitudinal analysis better addressed the adverse side-effects of prescription sleep medication in breast cancer survivors in real-world practices.

Certain limitations must be considered. We did not have complete data on BMI, and those with lower BMI might be more likely to have fractures. However, BMI may not be correlated with sleep medication use, thus the effect of this confounder might be minimal. Based on randomized clinical trials data that favored AIs over tamoxifen, physicians might have preferentially prescribed AIs to older patients, or to those with concerns for thromboembolism [17]. We focused our study on breast cancer survivors because they may be more likely to experience bone loss and fractures than women of similar ages in the general community for several reasons. These may include low levels of estrogen due to menopause induced by adjuvant cancer treatments, or due to the contraindication of prescribing postmenopausal hormone therapy to breast cancer survivors [14]. Combined with the adverse cancer treatment effects on bone health, breast cancer patients using hypnotic drugs may be even more susceptible to fracture risk. In a meta-analysis of 10 studies of older patients without cancer of both genders (age > 60 years), the fracture risk associated with the use of hypnotic drugs was also elevated (OR = 1.28; 95% CI: 1.08–1.53) [18]—although not as high as some of the effect measures observed in the present study (Table 5, Supplemental Table S2). Also, even though we only had spine bone mineral density on a subset of subjects (*n* = 8,498), the association between fracture risk and sleep medications persisted—the risk of fractures was greatest in those with worse T-scores indicating bone problems. Although we adjusted for adjuvant endocrine therapy (AIs and tamoxifen) and examined a comprehensive set of covariates, including time-varying sleep medications, antidepressants, anti-anxiety drugs, residual confounding cannot be precluded in our study.

In summary, we determined that almost 40% of breast cancer survivors had used sleep aid medications and its use increased the risk of fractures by 33%. This study bridged the gap between studies that examined this question in other vulnerable groups such as older women, but did not specifically include breast cancer survivors. Thus, clinicians must consider the safety of prescribing sleep medications in this vulnerable group. Our findings are also relevant to medical oncologists because they are increasingly providing general care to their patients [19, 20]. Given sleep medications strong association with fractures and that breast cancer survivors taking AIs are a susceptible given the AI’s adverse bone effects, another implication of this study is that sleep problems might be better resolved by non-pharmacologic approaches such as cognitive behavioral therapy, increasing daytime physical activity, and limiting caffeine and alcohol.

## Conclusion

Our study demonstrates a large fraction of breast cancer survivors (40%) used prescription sleep medications, and the most common was lorazepam, a benzodiazepine. Given that new users of sleep medications had a 40% increased fracture risk, even after adjustment for bisphosphonate use, caution is warranted when treating breast cancer survivors for sleep disturbance. Non-pharmacologic therapy may be considered to manage the sleep disturbances in breast cancer survivors that arise from distress or due to the side-effects of cancer treatments.

## Supplementary Information

Below is the link to the electronic supplementary material.

Supplementary file1 (docx 186 kb)

## Data Availability

Inquiries for a de-identified analytic dataset can be made to writing with the corresponding author. Data use agreements will be needed between KPSC and the requesting institutes as well as KPSC IRB review.
