# Supplementary material for: Sleep medication use and risk of fractures in breast cancer survivors
Source: Breast Cancer Res Treat. 2021 Sep 29;190(3):541–8. doi: 10.1007/s10549-021-06392-4 (PMC8558269; doi:10.1007/s10549-021-06392-4)
Supplement: Supplementary file 1 — Supplementary file1 (docx 186 kb) [file 10549_2021_6392_MOESM1_ESM.docx]

**SUPPLEMENTAL TABLES**

**SUPPLEMENTAL TABLE 1**: ICD-9 and ICD-10 codes for conditions

|  | **ICD9** | **ICD10** |
| --- | --- | --- |
| Fracture | 733.81, 733.82, 805, 808,813, 814-818, 819-824, 827, 828, 905.2-905.4, V54.10, V54.12-V54.16 | S12, S22, S32.0- S32.2, S32.3-S32.9, S52.x, S62, S72.x, S82.x |
| Forearm | 733.81, 733.82, 813, 819, 828, 905.2, V54.10, V54.12, | S52 |
| Femur | 820-821, 905.3, 905.4, V54.13, V54.15 | S72 |
| Lower leg including ankle | 822-824, 827, V54.14, V54.16 | S82 |
| Wrist and hand | 814-818 | S62 |
| Vertebral | 805 | S12, S22, S32.0- S32.2 |
| Pelvic | 808 | S32.3-S32.9 |
|  |  |  |
| Osteoporosis | 733.0x | M81.0, M81.6, M81.8 |
|  |  |  |
| Major depression | 296.2. 296.20-296.25, 296.26, 296.3, 296.31-296.34, 298.0, 300.4, 311 | F32.0-F32.5, F32.9. F33.0-F33.3, F43.40-F43.42, F33.9, F34.1 |
| Anxiety | 293.84, 300.00, 300.01, 300.02, 300.09, 300.10, 300.20, 300.21, 300.22, 300.23, 300.29, 300.3, 300.5, 300.89, 300.9, 308.0, 308.1, 308.2, 308.3, 308.4, 308.9, 309.81, 313.0, 313.1, 313.21, 313.22, 313.3, 313.82, and 313.83 | F06.4, F40.0-F40.2, F40.8, F40.9, F41.0-F41.9, F42.2, F42.3, F42.8, F42.9, F43.0, F43.1, F44.9, F45.8, F48.8, F48.9, F93.8 |
| Sleep disorders | 780.5X | G47.9 |
| Sleep apnea | 327.23 | G47.30 |
| Dementia | 294.2 | F03.90 |
|  |  |  |
| Hypertension | 401-405 | I10, I11.0, I11.9, I12.0, I12.9, I13.0, I13.10, I13.11, I13.2, I15.0, I15.1, I 15.2, I15.8, I15.9, I16.0, I16.1, I16.9, N26.2 |

**SUPPLEMENTAL TABLE 2:** The association of sleep medications use and risk of fractures among breast cancer survivors in the subset with spine bone mineral density (BMD) (n=8,498)
